# THE HEART OF DAILY LIFE: THE INTERPLAY BETWEEN EVERYDAY AFFECTIVE STATES, DAILY EVENTS, AND SUBJECTIVE BELIEFS

**DOI:** 10.1093/geroni/igae098.2105

**Published:** 2024-12-31

**Authors:** Patrick Klaiber, Theresa Pauly, Stacey Scott

**Affiliations:** Chair; Co-Chair; Discussant

## Abstract

Affective experiences profoundly shape well-being and quality of life throughout adulthood. They are intricately intertwined with social engagement and daily experiences like stressors and positive events. Individuals’ internal beliefs regarding aging and emotions can either facilitate or impede their engagement with daily events, and further impact emotional responses and regulation. Moreover, fluctuations in affect resulting from these daily experiences may cumulatively influence long-term mental and cognitive health outcomes. This symposium illuminates these dynamics by utilizing a set of daily diary studies using lifespan and older adult samples. The first presentation by Witzel et al. connects experiencing more frequent positive events and more favorable affective reactions to these events with better cognitive health. Klaiber and Pauly find that on days on which people feel older than usual, they experience more stressors, and fewer positive events, but also tend to show more favorable affective responses to these positive events. Thirdly, Pauly shows that positive affect mediates the association between social interaction time and decreased subjective age. Finally, Rutledge and Barlow shed light on differential associations between emotion beliefs and affect in younger vs. older individuals. Together, these studies offer valuable insights into the complex interplay between daily experiences, beliefs, affective fluctuations, and long-term health outcomes in adulthood. Stacey Scott will integrate the insights gained from these four papers, discuss their potential and limitations, and consider directions for future research.

